# The Effect of Everyday-Life Social Contact on Pain

**DOI:** 10.2196/53830

**Published:** 2024-04-30

**Authors:** Martin Weiß, Marthe Gründahl, Annalena Jachnik, Emilia Caya Lampe, Ishitaa Malik, Heike Lydia Rittner, Claudia Sommer, Grit Hein

**Affiliations:** 1 University Hospital Würzburg, Center of Mental Health, Department of Psychiatry, Psychosomatic and Psychotherapy, Translational Social Neuroscience Unit Würzburg Germany; 2 University Hospital Würzburg, Center for Interdisciplinary Medicine, Department of Anaesthesiology, Intensive Care, Emergency and Pain Medicine Würzburg Germany; 3 University Hospital Würzburg, Department of Neurology Würzburg Germany

**Keywords:** social contact, pain, ecological momentary assessment

## Abstract

Pain is a biopsychosocial phenomenon, resulting from the interplay between physiological and psychological processes and social factors. Given that humans constantly interact with others, the effect of social factors is particularly relevant. Documenting the significance of the social modulation of pain, an increasing number of studies have investigated the effect of social contact on subjective pain intensity and pain-related physiological changes. While evidence suggests that social contact can alleviate pain, contradictory findings indicate an increase in pain intensity and a deterioration of pain coping strategies. This evidence primarily stems from studies examining the effect of social contact on pain within highly controlled laboratory conditions. Moreover, pain assessments often rely on one-time subjective reports of average pain intensity across a predefined period. Ecological momentary assessments (EMAs) can circumvent these problems, as they can capture diverse aspects of behavior and experiences multiple times a day, in real time, with high resolution, and within naturalistic and ecologically valid settings. These multiple measures allow for the examination of fluctuations of pain symptoms throughout the day in relation to affective, cognitive, behavioral, and social factors. In this opinion paper, we review the current state and future relevance of EMA-based social pain research in daily life. Specifically, we examine whether everyday-life social support reduces or enhances pain. The first part of the paper provides a comprehensive overview of the use of EMA in pain research and summarizes the main findings. The review of the relatively limited number of existing EMA studies shows that the association between pain and social contact in everyday life depends on numerous factors, including pain syndromes, temporal dynamics, the nature of social interactions, and characteristics of the interaction partners. In line with laboratory research, there is evidence that everyday-life social contact can alleviate, but also intensify pain, depending on the type of social support. Everyday-life emotional support seems to reduce pain, while extensive solicitous support was found to have opposite effects. Moreover, positive short-term effects of social support can be overshadowed by other symptoms such as fatigue. Overall, gathering and integrating experiences from a patient’s social environment can offer valuable insights. These insights can help interpret dynamics in pain intensity and accompanying symptoms such as depression or fatigue. We conclude that factors determining the reducing versus enhancing effects of social contact on pain need to be investigated more thoroughly. We advocate EMA as the assessment method of the future and highlight open questions that should be addressed in future EMA studies on pain and the potential of ecological momentary interventions for pain treatment.

## Pain

Pain is associated with impaired physical and mental health and reduced quality of life [1,2]. The International Association for the Study of Pain defines pain as “an unpleasant sensory and emotional experience associated with actual or potential tissue damage”. Moreover, many pain disorders happen in the absence of tissue damage or any obvious pathophysiological cause [3]. Acute pain is an injury signal and usually recedes when the cause has resolved. Chronic pain is defined as lasting for a minimum of 12 weeks or longer than the expected healing time of an injury. The global prevalence of chronic pain is estimated between 20% and 30% of the population [4-6]. Thus, for many among us, pain is a frequent experience in daily life [2].

The prevalence and consequences of pain call for adequate scientific and clinical measurements and interventions. Yet, there is still a noticeable lack of efficient pain treatments. Among others, this has been traced back to insufficient pain monitoring [6,7] and heterogeneity regarding core outcome measures [8,9]. Pain assessments indicate the severity or quality of pain, enable a diagnosis, and provide indications for medical or therapeutic treatments and their effectiveness [9,10]. The most common pain measure is subjective reports of average pain intensity levels across a predefined period, for example, assessed via visual analog or numerical rating scales [10-12]. However, average and single-time pain measures overlook that (chronic) pain is dynamic rather than static and is characterized by inter- and intraindividual fluctuations in pain intensity, maximum pain levels, and related impairments, which are in turn associated with changes in cognition, affect, behavior, and motivation [2,10,13]. Intraindividual temporal pain variations can be important indicators of pain manageability and overall impairment, and their assessment can thus enhance the understanding and treatment of the pathophysiological, behavioral, and emotional processes related to pain [14,15]. Repeated, fine-grained, temporally precise, and longitudinal pain assessments in a real-life context are needed to adequately and representatively capture the structural and dynamic process of pain [10,13].

## Social Contact and Pain

Pain is multifaceted, and pain-related illnesses have heterogeneous symptoms, recoveries, and risks that can strongly differ between individuals [15]. While some patients’ daily lives are highly disrupted and impaired, others struggle less in coping with pain. Factors that contribute to these differences are yet poorly understood [15,16]. Researchers are therefore calling for the inclusion of facets other than pain intensity and variability into pain-related research [17]. Notably, pain is often described as a biopsychosocial phenomenon, referring to the interplay between physiological pain-related processes and psychological and social factors [18]. Previous pain studies have shown that pain can impair our social relationships and social functioning [13], for example, by reducing our participation in social life [19,20]. This may in turn increase pain intensity and related symptoms and impairments (eg, negative cognitive processes and impaired quality of life) [2,18]. However, “positive” psychosocial experiences can also decrease pain, for example, social interactions providing social support [1,21]. Social contact has thus been related to either reductions or increases in pain experiences, depending on factors such as the provision of adequate versus extensive social support [22,23], supportive versus unsupportive behavior by others [24,25], or characteristics of the social partners (eg, outgroup vs ingroup membership; sex: female vs male) [26,27]. Protective effects of social contact on pain-related responses are evident in reducing influences on the physiological stress system (“social buffering”) [28,29], but also on cognitive and emotional facets such as negative thoughts and negative affect. Consequently, social contact can lead to improved pain reappraisal, less pain-related thoughts, and lower perceived pain intensity [1] and could therefore be an important chess piece in both the development and treatment of pain. Yet, only a few studies have examined how social contact affects the development, resolution, or persistence of pain in a daily life context.

In this paper, we review the status quo of social pain research in daily life and advocate ecological momentary assessments (EMAs) as the assessment method of the future. In particular, we focus on the question of whether everyday-life social support reduces or enhances pain, as laboratory studies provide evidence for both effects. The first part of the paper provides a general overview of the use of EMAs in pain research. Next, we summarize the results of these studies on everyday-life social contact on pain and discuss the ambiguous relationship between social support and pain in more detail. Finally, we highlight open questions and point out future directions.

The literature research for this opinion paper was conducted in June and July 2023 in the databases Google Scholar and PubMed. Searches included the topic “pain” and terms representing EMA, that is, “ecological momentary assessment” OR “ambulatory assessment” OR “experience sampling” OR “diary assessment” OR “intensive longitudinal method” OR “intensive longitudinal study” OR “real-time” OR “daily life” OR “everyday life.” The search results were limited to papers that were written in English. The database searches were complemented with manual reviews of the reference lists of relevant papers.

## Use of EMAs

EMA (also known as experience sampling or ambulatory assessment) has become increasingly popular in psychological and clinical research over the last decades. In parallel, daily diaries are used as part of clinical diagnostics, as they can provide relevant information (eg, pain intensity ratings) in patients’ everyday life. The compliance with daily diaries is comparable to EMAs (approximately 85%) [30,31]. Daily diaries are occasionally categorized among EMA methods [32]. However, they are usually completed only once or twice per day at fixed time points. In contrast, (more advanced) EMA has the capacity to capture various aspects of human behavior and experiences multiple times a day, in real time, in high resolution, and in naturalistic and ecologically valid settings [33-35]. These multiple measures of daily-life experiences can be put into relation to fluctuations of symptoms (eg, pain) throughout the day, depending on affective, cognitive, behavioral, and—last but not least—social factors [12,36,37]. As ecological momentary interventions (EMIs), EMA methods can even be used for interventional purposes [12,38] and provide treatment once pain levels increase (eg, provision of social support) [39].

EMA offers important advantages to (social) pain research. It reduces the problem of recall bias and thus “purifies” pain measurements, as it captures current experiences rather than retrospective memories [10,40]. Compared with single-time assessments, EMA is less prone to social desirability, cognitive biases, and measurement error [41]. It provides flexibility regarding the selection of representative sampling schedules and intensities, which can be tailored to the study objectives and the demands and capability of specific (pain) samples [17]. In accordance with this, compliance with EMA in patients with different pain conditions was high in previous research [12,42,43]. A key advantage for pain research in particular is EMA’s ability to observe and summarize within-person effects and temporal dynamics, including time-lagged relationships, through repeated, longitudinal measurements [17,34]. As summarized by Stone et al [17], EMA can quantify, predict, and potentially influence the ebb and flow of pain, for example, after surgery [44]. What is more, its real-world setting (eg, everyday-life social contact) maximizes the ecological validity of pain-related experiences [17]. Thus, EMA can measure daily-life pain several times a day [13,45], for weeks [9], or even months [46] and capture its relation to daily-life experiences such as social contact [22], work-related stress [47], physical activity [48], and countless other potential influences on pain [17].

EMA can further detect differences between pain conditions, for example, higher variability in fatigue levels in women experiencing fibromyalgia compared with women with rheumatoid arthritis and osteoarthritis [49]. The variability in pain symptomology, related impairments, and treatment response among patients with pain calls for personalized treatments, and continuous, ecological, and momentary pain assessments can advance the design and validation of such interventions [6,50]. EMA could advance the development and monitoring of preventive interventions [51] by observing pain levels of those at risk (eg, patients with acute pain) over time, but also in relation to potential influences in daily life. In the wake of a shift away from on-demand medication and a strong focus on pain in pain treatment, EMA could contribute to tailoring basic analgesia to individual patient needs and patterns [52-54].

Despite these advantages, EMA application in pain research is still relatively rare and, regarding its methodological choices, heterogeneous, unclear, and often outdated [12,17]. By 2020, at least 116 studies had applied EMA for the measurement of pain [12,17]. May et al [12] reviewed 62 research projects that were reported in 105 papers. Only 9 projects (14.5%) used smartphones for data collection, while the majority (39/62, 63%) used other electronic methods, except for phone calls (3/62, 4.8%) and paper booklets (11/62, 17.7%). Stone et al [17] updated this review, focusing on EMA papers published in *PAIN* and *the Journal of Pain* between 2016 and 2020. In addition to those reported in May et al [12], they found 11 papers covering 9 projects, including 2 (22%) projects using smartphone applications and 6 (67%) projects using other full electronic data assessment tools (eg, personal digital assistants such as palmtop computers). Notably, several pain EMA studies used obsolete instead of modern EMA measurement tools such as smartphone-based surveys (eg, telephone calls, pen and paper, or handheld computers) [25,55]. This is a pity, as there is a high acceptance and feasibility of smartphone-based assessments [56-58]. Smartphones are an easy-to-use and broadly available measurement tool with diverse and accurate digital data collection possibilities that almost everyone in our modern world is familiar with [14,59]. Today’s technological innovations even enable clinicians to receive in-time feedback on their patients’ current (pain) experiences, which could be used for timely interventions. This approach can be integrated into EMIs to deliver individualized, momentary treatments in dependence on current pain-related experiences (eg, predictors of higher pain levels), even without the active participation of a clinician [17]. The prospects and findings outlined above raise the questions of why (social) pain research and practice have not yet shifted to a broader application of (modern) EMA.

## Pain and Social Contact in Daily Life

Within and beyond pain research, EMA seems particularly relevant when investigating the effects of an integral part of our daily lives: social contact [1,60]. Notably, social contacts are diverse as they differ in factors like length, content, aim, and tone, but also regarding the number, characteristics, and relationships of social partners [61-63]. Only intensive and ecologically valid measurement tools can adequately capture this diversity. Although the influence of social contact on pain has been acknowledged in the literature [1,21], very few studies have investigated this interplay in daily life settings [22,64,65].

The existing EMA studies on pain and social contact provide promising first insights (see Table 1 for an overview). For example, pain-related social impairment in daily life was evident in a study with 102 adults with multiple sclerosis using wrist-worn monitors. The monitors were worn on the nondominant hand, except during activities such as showering, bathing, or swimming. Participants were asked to rate acute pain, fatigue, depressive mood, and cognitive function on a scale of 0 to 10, 5 times a day using a wrist-worn device [50]. In addition, participants were asked to provide a more detailed report on their social participation with a web-based survey once per day in the evening. However, to uncover relevant features of social contact, researchers usually present several questions to retrieve more comprehensive impressions of the features of the contact (eg, quantity of interaction partners, number of strangers, familiarity and gender of the interaction partner, or perceived personality traits) [31,60,66]. Thus, wrist-worn devices might not be as suited as smartphones when it comes to extensive social contact research in daily life as presenting multiple-choice EMA questions is more burdensome compared with smartphones [67]. In the study described above, higher pain was related to lower same-day social participation [50]. Other EMA results imply ambiguous effects of social contact on pain. In a small-sampled study with older adults with HIV (n=20) and smartphone surveys (5/day for 1 week), social activity was related to higher levels of fatigue and pain during the day, but also to higher levels of happiness. Looking at temporal relations, higher pain was related to previously being alone, but also to a higher likelihood of not being alone later during the day [65].

**Table 1:** Selection of studies exemplifying ecological momentary assessment research differing in targeted pain syndrome, measuring method, sample size, social contact measures, and results.

Study	Pain syndrome^a^	Measuring method	Sample size, N	Social contact measure	Results
Kratz et al [50] (2017)	Multiple sclerosis	Wrist-worn monitor	102	“doing all of the family activities that I want to do” “doing all of the activities with friends that are really important to me” “doing all the leisure activities with others that I want to do” “doing all of the work that I feel I should do (include work at home)”	Higher pain related to lower same-day social participation
Paolillo et al [65] (2018)	HIV	Smartphone surveys	20	“Who is with you at this moment?” “Since the last alarm, how many times did you socialize with someone else (e.g., spent more than 5 min talking or communicating with someone else)?”	Social activity related to higher levels of fatigue, pain, and happiness Higher pain related to previously being alone and a higher likelihood of not being alone later on
Herbert et al [64] (2022)	HIV	Smartphone surveys	66	“Since the last alarm, how many times did you socialize with someone else [e.g., spent more than five minutes talking/ communicating with someone else]?”	Higher frequency of recent social contact related to lower current pain Higher current pain was linked to a decrease in subsequent social interactions Higher current negative affect related to higher current pain; this relationship was buffered by increased recent social contact
Rivera et al [68] (2020)	Osteoarthritis of the knee	Telephone calls	268	“open ended question...asking the participants to indicate what they were doing just prior to receiving the phone call if an interaction was occurring...type of interaction [was assessed] from ‘positive’ to ‘negative’, with remaining categories consisting of ‘help given’, ‘help received’, ‘neutral’, and ‘professional’.”	In general, more social interactions reduced the association between pain and negative affect On a within-day level, more social interactions related to more positive affect

^a^Pain syndrome: pain-related disease of study participants.

In a larger sample of older adults with HIV (n=66) and smartphone surveys (4/day for 2 weeks), Herbert et al [64] sought to replicate the results reported by Paolillo et al [65], showing an association between recent social contact and lower current pain. In contrast, Herbert et al [64] found that higher current pain was temporally associated with less subsequent social contact. Interestingly, recent social contact buffered the relation between negative affect and current pain, as only those with low contact frequency exhibited an increase in pain with higher negative affect.

A study with 268 adults with osteoarthritis of the knee investigated the impact of social interactions and pain on daily affect via telephone calls (4/day for 1 week). On a general level, more social interactions reduced the association between pain and negative affect. On a within-day level, more social interactions were related to more positive affect. These results are limited by the minimalistic assessment of daily social interactions: the occurrence of social interactions was coded based on a general question regarding the participant’s activity prior to receiving the phone call, and no additional social aspects were assessed [68]. However, similar to other health-related contexts (eg, anxiety-related responses) [60], daily-life pain might change in dependence on the personal characteristics of social partners, such as their gender or familiarity. For instance, there are indications from the laboratory that social support by strangers is less efficient in reducing pain than social support provided by more familiar social partners [21,69]. Similarly, female social partners tend to provide more care and may thus have more pain-reducing effects [70]—or, if providing too much care, pain-enhancing effects (see below). Such influences have rarely been explored in pain research but could be highly relevant for daily-life pain.

Importantly, EMA can expand the researcher’s gaze to the experiences and behaviors of others that are part of an individual’s social environment. Interview-based research has shown that chronic pain also impacts a patient’s relatives and partners, who for example report changes in leisure activities, sleep disturbances (n=12) [71], feelings of powerlessness, alienation, and emotional distress (n=9) [72]. This can in turn impair the relationships between patients and their social networks [72], thus increasing pain symptoms [73]. It is therefore important to understand when and how pain affects the social environment and vice versa. For instance, interviews with 27 patients with chronic low back pain and their partners indicated that partners’ pain responses can be interpreted differently by patients and partners, resulting in misinterpretations. Thus, the interpretation of pain responses may determine whether the behavior in question increases or decreases the patient’s pain experiences [74]. Based on these findings, experiences from the family’s perspective provide additional insight into factors affecting the development, maintenance, or resolution of pain while also providing indications for intervention. A more fine-grained EMA assessment in daily life, ideally with parallel assessments in patients and their social environments as well as with bigger sample sizes, would be particularly valuable here.

## Pain and Social Support: An Ambiguous Relationship

One social factor particularly associated with changes in pain intensity is social support [75,76]. Social support can be characterized as the experience of feeling supported, cared for, and connected to others, contributing to a sense of belonging [77]. Researchers distinguish between perceived and actual (or received) social support [78]. While perceived social support is an individual’s subjective evaluation of the emotional and psychological support they believe is available from family and friends in times of need, received social support quantifies the actual support received. Importantly, both types of social support are only moderately correlated [79,80], as the amount of support someone receives does not always align with how much they feel they are supported (for meta-analysis, see [79]). In general, social support is associated with positive emotional states and more effective pain adaptation, which can predict lower pain and improved psychological functioning [81]. It can serve as a coping mechanism, enhance the ability to cope with pain, and facilitate pain management [1,24]. Insufficient and absent social support and social integration have been related to increases in pain [73,82] and even emerged as antecedents or magnifiers of chronic pain [83,84]. Contrasting these pain-buffering effects of social contact, some findings imply the pain-enhancing effects of social contact and support. For instance, a social partner’s extensive pain-related concerns in relation to painful stimulation [85] or chronic pain experiences [86,87] can lead to increased pain-related experiences. Solicitous responses (eg, the encouragement to be less active) and negative responses (eg, the expression of frustration or anger about pain) by significant others were previously related to higher pain and disability in patients with chronic pain [86,88,89].

In line with these negative outcomes, operant and cognitive-behavioral models of pain [90,91] assume that people experiencing pain communicate their pain to close others through pain behaviors and that others’ (particularly romantic partners’) emotional or solicitous responses reinforce these behaviors, while negative responses punish and thus reduce future pain responses [92]. Consequently, the models suggest that social support may compromise self-sufficiency, reinforce maladaptive pain behaviors, foster dependency, and interfere with a patient’s ability to cope with pain [24,93]. In contrast, interpersonal process models [94] assume that empathic and validating pain-related responses by spouses lead to positive outcomes by enhancing intimacy and emotion regulation, while negative responses lead to negative pain-related outcomes [25,92]. In relation to these models and partial evidence for each of them, Mogil [23] has suggested a U-shaped relationship between social support and pain experience: in general, the perception of social support versus no support decreases pain [1], but extensive solicitous concern may enhance pain intensity and related factors such as functional disability and pain catastrophizing [95,96] and foster pain expression in patients with pain [87,93].

EMA has provided the first steps to respond to these relevant yet contrasting assumptions with closer-to-life data. In 109 men living with HIV, smartphone-based EMA across 1 week (3 assessments per day) showed that social support related to lower subsequent pain intensity on a within-person level. Results further suggest that this relation may be moderated by between-person factors such as attachment-related avoidance, which was associated with higher pain reduction through social support [22]. In a study of older adults experiencing arthritis, the researchers distinguished between the effects of emotional, solicitous, and negative support. The pain was lower after having received emotional support from one’s spouse. However, solicitous support (eg, expression of pain-related concern and comfort) and avoidance behavior by one’s spouse were associated with higher pain levels [25]. While highly interesting, this EMA study investigated a very specific sample and used outdated methods (telephone interviews 2 times a day) instead of more feasible and fine-grained smartphone surveys. Similarly, operant models have been particularly investigated in chronic pain couples [25,86,87]. Previous findings should be validated and extended by research with modern methods, diverse pain samples, and other social network members (eg, friends and children).

Note that some research could not find a relationship between social support and pain [97,98]. For example, an EMA study investigating factors contributing to the chronification of pain after potentially traumatic injuries used text message–based questionnaires for 2 weeks in 67 adults. They did not find a relationship between social support and pain on a daily level. However, the study only included 1 assessment per day, and a more fine-grained (and modern) assessment may have provided more insight, as also stated by the authors [98].

In sum, both the effects of social contact on pain, as well as pain-related social impairment, seem to differ within and between days and individuals depending on numerous factors such as pain syndromes, temporal relations, the nature of social interactions, or the social partners and their experiences. A lack of social contact may enhance pain, but it remains unclear under which conditions pain reduces [50] or fosters [65] social participation. In some, but not all, pain syndromes, short-term symptom-reducing effects of social contact (eg, on fatigue) may become overshadowed by next-day increases in fatigue [99]. Obtaining and integrating experiences from a patient’s social environment can provide additional information, for example, regarding the misinterpretation of pain behaviors [74]. Finally, factors determining enhancing versus reducing effects of emotional and solicitous social support need to be investigated more thoroughly [22,25].

## Future Directions

There are still some challenges in the EMA application that remain unsolved, as recently pointed out by Stone et al [35]. Despite reports of high feasibility [14,57,58], acceptance [11], and compliance [12,42], EMA can be burdensome to patients with pain [43]). We should therefore strive to make EMA as convenient and appealing as possible, for example, by low weight and size of the assessment tools, easy-to-use interfaces, appealing designs, gamification, or motivational incentives [11,41,100]. There are also issues to be considered from the researchers’ and practitioners’ side. For instance, EMA protocols require thorough planning and execution, and complex EMA data sets require adequate statistical analyses [33,101]. Other issues include the accessibility of EMA tools, which are currently often limited by factors such as smartphone operating systems (there is a lack of EMA tools running on iOS), and the provision of data protection, particularly when using private smartphones as assessment tools [102]. Moreover, there is a potential selection bias of EMA respondents or the question of whether the interpretation of EMA survey questions is congruent between and within participants (and in accordance with the researcher’s intentions) [35]. Solving and standardizing these issues could further advance the field of EMA research [35].

Furthermore, one could inquire whether EMA and its items influence individuals’ behavior in social interactions or introduce biases in the perception of their interaction partners. To address this intriguing question, studies would need to assess whether the quality and quantity of social interactions change during the EMA assessment period, that is, with increasing numbers of prompts. This would be an interesting issue for future research. Another relevant aspect of EMA that might be affected by the context of social contact is the participants’ compliance [103]. Among the 69 projects involving patients with chronic pain from the review by May et al [12], 39 provided data on completion rates. These projects exhibited an average completion rate of 86%, with individual project rates ranging from approximately 29% to 99%. EMA research examining social interactions with large and healthy samples (≥115 participants) also showed high completion rates of at least 85% [30,31]. Notably, participants in these studies received monetary compensation supplemented by additional bonuses for responding to each prompt or for exceeding a specific number of answered prompts, which may have enhanced compliance. In line with this, a review of pain studies with electronic diaries [42] observed that financial incentives as well as shorter diaries contribute to higher compliance rates (83%). Future research using smartphone-based EMA to study social interactions in patients with pain should thus consider the use of shorter surveys as well as financial incentives per prompt or prompt threshold, contingent upon the availability of funding and ethical approval for compensating patients, to maintain and enhance compliance.

Overall, more homogenous EMA pain study protocols and measurements would strengthen future EMA pain research. As pointed out in a review, previous EMA studies frequently lack detailed reports of factors such as study design decisions and completion rates. They use heterogeneous pain scales, for example, regarding scale labels and points [12]. This decreases the comparability and generalizability of EMA-based results. Future research should aim for a homogenous pain scale to enhance comparability between studies. Numeric rating scales (most commonly anchored from 0 to 10, eg, with 0=“no pain” and 10=“pain as bad as you can imagine” or “worst pain imaginable”) [15,104] are popular, fine-grained, and insightful [10,12] and could serve as a common tool. Based on the experience of our and others’ previous research on social interactions, we recommend the consideration of several contextual factors in order to obtain clean and interpretable EMA data [31,38,60,105]: Was the interaction face-to-face or not? How many persons were involved in the interaction? Are questions about an interaction partner’s characteristics targeting a single person or the group? How long is or was the interaction? Is it currently ongoing or, if not, how long ago did it end? Up to what time interval between social contact and its assessment should the data be analyzed to avoid recall bias? Are attention checks needed to ensure clean data, particularly in long-term surveys? and Should an alternative, equally long questionnaire be presented in the absence of social contact to prevent participants from avoiding the survey to save time?

It is also noteworthy that a vast majority of previous social and pain-related EMA studies have missed the opportunities of combining subjective momentary pain reports with ambulatory physiological assessments of autonomic measures (eg, heart rate measured with electrocardiogram sensors) and physical activity (eg, through accelerometer sensors). Ambulatory sensors may provide additional information on pain intensity, change, and interference in daily life. There are numerous technologically advanced measurement tools for assessing physiological measures associated with pain experiences and related factors, including wearable electrocardiogram sensors and smartwatches with biosensors. Such ambulatory measurements are objective, unbiased, and can collect data continuously and at a high resolution [33,41]. Previous research has already used ambulatory sensors to investigate social interaction and support in daily life [60,106]. The acceptance and feasibility of mobile measurement devices such as smartwatches are high among the general population [41,107], and the technology is quickly evolving to provide even more opportunities. Next to simply assessing physiological correlates of subjective pain experiences, wearable devices could also be implemented to detect physiological changes and impairments associated with a specific pain syndrome and consequently trigger prompt interventional steps for patients with pain. For instance, in a similar approach as reported by Kim et al [108] on depression, physical activity patterns tracked with ambulatory sensors could be used to objectively estimate momentary pain levels. Combining smartphone-based pain surveys and portable sensors to measure physiological responses (eg, heart rate, heart rate variability, or other indicators of stress) could provide a more profound understanding of the contextual and proximal causes and consequences of pain and thus advance pain treatment [2,41].

## Outlook

This opinion paper requests a drastic expansion of EMA-based pain research, particularly in the context of social contact. The first steps have been made, yet much more remains to be explored regarding the social-pain phenomenon. EMA should not be the sole source of pain-related data, however. Rather, researchers and clinicians should complement clinical assessments (eg, average pain assessments, pain questionnaires) and treatments with EMA as well as other methodological approaches [10,11], such as brain imaging [109], physiological sensors to capture objective measures such as blood pressure or physical activity [33,41], or data from biosamples such as genetics, transcriptomics, and proteomics [110,111]. Moreover, EMI provides great potential for pain research and practice. Future directions may include the personalization of smartphone-based treatments with more extensive and more convenient assessment and treatment tools [11], for example, with respect to the interindividual differences in factors such as pain variability and function [11] or the potential influence of daily-life surroundings such as social contacts [25,64]. Such just-in-time adaptive interventions [112] could help patients with pain identify associations between their current pain level and their momentary activity [113].

## Abbreviations

EMA: ecological momentary assessment
EMI: ecological momentary intervention
